# *Streptococcus suis* Meningitis in Swine Worker, Minnesota, USA

**DOI:** 10.3201/eid1902.120918

**Published:** 2013-02

**Authors:** Heather N. Fowler, Paul Brown, Albert Rovira, Beth Shade, Kathryn Klammer, Kirk Smith, Joni Scheftel

**Affiliations:** Author affiliations: Minnesota Department of Health, St. Paul, Minnesota, USA (H.N. Fowler, B. Shade, K. Klammer, K. Smith, J. Scheftel);; Lakeview Medical Clinic, Waconia, Minnesota, USA (P. Brown);; University of Minnesota, St. Paul (A. Rovira)

**Keywords:** Streptococcus suis, bacteria, bacterial meningitis, meningitis, swine, streptococci, Minnesota

**To the Editor:** *Streptococcus suis* is a major bacterial pathogen in swine worldwide. Historically, cases in humans have occurred sporadically, mostly in Asia (*1*,*2*). However, an outbreak in China involved 215 human cases and 39 deaths (*3*). Only 3 human cases of *S. suis* disease were documented in the United States before 2011: 2 domestically acquired cases in New York and Hawaii, and 1 case in a person in California who was probably exposed in the Philippines (*4*). We describe a case of *S. suis* disease in a swine worker in Minnesota, USA.

The case-patient was a previously healthy 60-year-old man (truck driver). On December 14, 2011, severe headache and chills developed, which he attributed to the onset of influenza. He had a history of migraine headaches, and used prescription medications to treat his headache. However, on December 15, he awoke with a severe headache that was unresponsive to treatment. Despite having to stop his truck several times because of the severe headache, he successfully completed his delivery route.

Early on December 16, his wife drove him to a nearby emergency department after he did not respond to ordinary commands. The patient had reduced coordination and behaved aggressively. His blood pressure was 92/52 mm Hg. He underwent intubation for 24 hours for airway protection, and a lumbar puncture was performed.

Cerebrospinal fluid (CSF) had a leukocyte count of 10,501 cells/μL (99% neutrophils), a protein level of 509 mg/dL, and a glucose level of 38 mg/dL. A few gram-positive diplococci were observed in CSF. Complete blood count showed a leukocyte count of 14,800 cells/μL (92% neutrophils), a hemoglobin level of 14.1 g/dL, and a platelet count of 157,000/μL. *Streptococcus suis* was isolated from CSF and 2 of 4 blood cultures. Identification of *S. suis* was confirmed by using 16S rRNA gene sequencing at the Minnesota Department of Health.

The patient was given decadron, ceftriaxone, ampicillin, vancomycin, and acyclovir. During hospitalization, antimicrobial drugs were tapered until he received only ceftriaxone. Major symptoms were severe headache and nausea. He was discharged in good condition on day 10 of hospitalization and then completed a 14-day course of ceftriaxone.

There are 35 known serotypes of *S. suis* (*1*). Of these serotypes, serotype 2 is most commonly identified in infected swine and humans (*2*). The *S. suis* isolate from this patient was identified as serotype 2 by coagglutination test at the International Reference Laboratory at the Université de Montréal (*5*). The sequence type was identified by PCR as type 25, a common type in North America (*6*,*7*). The isolate was positive by PCR for the gene encoding virulence-associated factor muraminidase-released protein and negative for genes encoding virulence-associated extracellular factor and suilysin (*8*). The isolate was genotyped by enterobacterial repetitive intergenic consensus PCR and compared with 750 swine isolates in the University of Minnesota Veterinary Diagnostic Laboratory database (*9*). The obtained fingerprint matched that of 15 *S. suis* isolates from swine meningitis cases in Minnesota and Indiana during 2006–2010.

The patient worked for a trucking company that transports swine throughout the Midwest. His daily work required traveling to swine farms in Minnesota and making occasional trips to South Dakota and Iowa. His job was to load slaughter-weight swine into the truck and deliver them to regional slaughterhouses. Approximately 1 month before illness onset, he reported moving swine from a farm on which the farmer reported pneumonia, a rare yet reported manifestation of *S. suis* infection in swine.

The patient reported always wearing coveralls, boots, and gloves while loading and unloading swine, but he wore a dust mask only occasionally. He had no recent foreign travel and no skin breaks. However, absence of open wounds has been noted in previous case-patients (*10*).

The reported incubation period for *S. suis* infection in humans ranges from hours to weeks, and open wounds are associated with shorter incubation periods (*2*). Case-patients in the United States reported known risk factors, including handling ill swine or slaughtering and processing swine for meat (*4*). In this instance, the patient only loaded and unloaded slaughter-weight swine from his truck. He reported transporting swine that had pneumonia, which is common in finishing stages of swine production. However, although *S. suis* can cause pneumonia, this disease in finishing swine is probably caused by other common pathogens such as *Pasteurella multocida, Mycoplasma hyopneumoniae,* influenza virus, and porcine reproductive and respiratory syndrome virus. A definitive source of infection for this patient was not identified. This case demonstrates a rare but potentially under-recognized occupational hazard for workers in the swine industry in the United States.
